# Supplementary material for: Carbon monoxide as a cellular protective agent in a swine model of cardiac arrest protocol
Source: PLoS One. 2024 May 15;19(5):e0302653. doi: 10.1371/journal.pone.0302653 (PMC11095756; doi:10.1371/journal.pone.0302653)
Supplement: Attachment — Submitted filename: Response to Reviewers.docx [file pone.0302653.s001.docx]

Please include the following items when submitting your revised manuscript:

- A rebuttal letter that responds to each point raised by the academic editor and reviewer(s). You should upload this letter as a separate file labeled 'Response to Reviewers'.
- A marked-up copy of your manuscript that highlights changes made to the original version. You should upload this as a separate file labeled 'Revised Manuscript with Track Changes'.
- An unmarked version of your revised paper without tracked changes. You should upload this as a separate file labeled 'Manuscript'.

If you would like to make changes to your financial disclosure, please include your updated statement in your cover letter. Guidelines for resubmitting your figure files are available below the reviewer comments at the end of this letter.

If applicable, we recommend that you deposit your laboratory protocols in [protocols.io](http://protocols.io/) to enhance the reproducibility of your results. Protocols.io assigns your protocol its own identifier (DOI) so that it can be cited independently in the future. For instructions see: <https://journals.plos.org/plosone/s/submission-guidelines#loc-laboratory-protocols>. Additionally, PLOS ONE offers an option for publishing peer-reviewed Lab Protocol articles, which describe protocols hosted on [protocols.io](http://protocols.io/). Read more information on sharing protocols at <https://plos.org/protocols?utm_medium=editorial-email&utm_source=authorletters&utm_campaign=protocols>.

**General Response**

Thank-you for the reviews that will strengthen this manuscript. We have carefully reviewed all the comments and revised accordingly. In particularly for this revision, we have made the following changes:

1. Made the recommended changes outlined in the journal checklist on the next page and respond to a particular point as needed
2. Addressed the journal checklist below

**Journal requirements checklist (make sure complete during the revisions):**

[x] 1. When submitting your revision, we need you to address these additional requirements.

Please ensure that your manuscript meets PLOS ONE's style requirements, including those for file naming. The PLOS ONE style templates can be found at 
<https://journals.plos.org/plosone/s/file?id=wjVg/PLOSOne_formatting_sample_main_body.pdf> and 
<https://journals.plos.org/plosone/s/file?id=ba62/PLOSOne_formatting_sample_title_authors_affiliations.pdf>.

[x] 2. Please amend your current title to include the word "protocol".

[x] 3. We note that the grant information you provided in the ‘Funding Information’ and ‘Financial Disclosure’ sections do not match.

When you resubmit, please ensure that you provide the correct grant numbers for the awards you received for your study in the ‘Funding Information’ section.

[x] 4. In your Data Availability statement, you have not specified where the minimal data set underlying the results described in your manuscript can be found. PLOS defines a study's minimal data set as the underlying data used to reach the conclusions drawn in the manuscript and any additional data required to replicate the reported study findings in their entirety. All PLOS journals require that the minimal data set be made fully available. For more information about our data policy, please see <http://journals.plos.org/plosone/s/data-availability>.

""Upon re-submitting your revised manuscript, please upload your study’s minimal underlying data set as either Supporting Information files or to a stable, public repository and include the relevant URLs, DOIs, or accession numbers within your revised cover letter. For a list of acceptable repositories, please see <http://journals.plos.org/plosone/s/data-availability#loc-recommended-repositories>. Any potentially identifying patient information must be fully anonymized.

Important: If there are ethical or legal restrictions to sharing your data publicly, please explain these restrictions in detail. Please see our guidelines for more information on what we consider unacceptable restrictions to publicly sharing data: <http://journals.plos.org/plosone/s/data-availability#loc-unacceptable-data-access-restrictions>. Note that it is not acceptable for the authors to be the sole named individuals responsible for ensuring data access.

We will update your Data Availability statement to reflect the information you provide in your cover letter.

[x] 5. We note that you have stated that you will provide repository information for your data at acceptance. Should your manuscript be accepted for publication, we will hold it until you provide the relevant accession numbers or DOIs necessary to access your data. If you wish to make changes to your Data Availability statement, please describe these changes in your cover letter and we will update your Data Availability statement to reflect the information you provide.

[x] 6. Please amend either the title on the online submission form (via Edit Submission) or the title in the manuscript so that they are identical.

[ ] 7. Please include your full ethics statement in the ‘Methods’ section of your manuscript file. In your statement, please include the full name of the IRB or ethics committee who approved or waived your study, as well as whether or not you obtained informed written or verbal consent. If consent was waived for your study, please include this information in your statement as well.

Response: We did include the following

“All procedures are currently approved by the Institutional Animal Care and Use Committee at the Children’s Hospital of Philadelphia (CHOP) and performed in accordance with the National Institutes of Health Guide for the Care and Use of Laboratory Animals for related studies with both CO and cardiac arrest in our lab.”

[X] 8. Please upload a copy of Figure 1, to which you refer in your text. If the figure is no longer to be included as part of the submission please remove all reference to it within the text.

[x] 9. Please review your reference list to ensure that it is complete and correct. If you have cited papers that have been retracted, please include the rationale for doing so in the manuscript text, or remove these references and replace them with relevant current references. Any changes to the reference list should be mentioned in the rebuttal letter that accompanies your revised manuscript. If you need to cite a retracted article, indicate the article’s retracted status in the References list and also include a citation and full reference for the retraction notice.

**Response to Reviewers**

**Reviewer #1:** Thank you for giving me an opportunity to review this manuscript. This manuscript describes a study protocol to investigate the feasibility of CO as a cellular protective agent in a pig model of cardiac arrest. Overall, the manuscript clearly presents the rationale and method of the study. I have only a few minor suggestions listed as follows.

Abstract
1. Abstract, Line 9. Please revise the term ‘post-cardiac patients’. (patients with cardiac arrest or patients resuscitated from cardiac arrest?)

*Response: We reviewed the manuscript and updated the term for post cardiac arrest patients to “**post-arrest patients” which is the proper term. We revised both the abstract and the main manuscript.*

2. Introduction, Line 7. It may be better to move the sentence “In one of our studies, we found that swine exposed to 2000 ppm for over an hour reliable COHb of around 50%.3” from the introduction section to the method or discussion section.

*Response: We move this sentence from the introduction to the methods as recommended by reviewer 1. We also restructured the introduction to focus more on cardiac arrest as well as the gaps related to the need for more therapies as opposed to starting with carbon monoxide poisoning that we feel flows better.*

3. Introduction. Please spell out all abbreviations including MI and CI in the first appearance. Please check all abbreviations throughout the manuscript.

*Response: We spell out all abbreviations such as MI (myocardial infarction) and CI (cardiac index) in the manuscript in tracked changes.*

4. Introduction. A more detailed description of why CO is a more ideal agent than NO or H_2_S is needed.

*Response: We have already described the differences between CO, NO and H_2_S in the discussion section that flows better here as opposed to placing in the introduction.*

*Discussion Paragraph 5*

*“Other agents with similar properties to CO have been investigated, including nitric oxide (NO) and hydrogen sulfide (H_2_S). H_2_S is also a toxic gas with overlapping properties to CO that also can be found in the gas industry and sewage handling. While H_2_S has been shown to have cardioprotective benefits, it is considerably more toxic than CO with a very low therapeutic index that makes clinical implementation challenging. Another gas that also has been used is NO that is produced endogenously with important biological functions that primarily involve vasodilation and angiogenesis. NO has clinically been used for the treatment of ischemic heart disease but also has been used post-arrest to reduce IR injury. Despite the potential benefits of NO, NO is reactive at higher doses that can cause increased ROS production leading to DNA damage and can also cause methemoglobinemia (MetHb). MetHb can worsen ischemia as the oxidized iron in the affected hemoglobin can no longer accept or deliver oxygen to tissue. Taken together, while H_2_S, NO and CO are all potentially poisonous, CO is not considered as reactive as NO and has considerably more robust experimental and clinical data supporting use of CO over H_2_S and NO with a more favorable safety profile.”*

5. It is necessary to highlight the necessity of this study in relation to previous studies that investigated the effect of CO administration after arrest.

*Response: We revised to better discuss the necessity of this study as there is a paucity of work in this area despite both in vitro and small animal work that support the of CO as a therapeutic in cardiac arrest.*

*Discussion Paragraph 3*

*“While there are some small animal models in this area, there is a paucity of large animal models that recapitulate what can occur in patients. Our prior work with swine demonstrates a much more translational model that better captures the physiology and biomolecular findings in patients. Leveraging our swine models of both CO exposure and cardiac arrest, positive findings from this study would allow more rapid clinical translation in the future.”*

Materials and methods
6. The double quotation marks in the first sentence in the animals and overall study design section are incomplete.

*Response: This has been fixed as recommended.*

7. Please provide a more detailed description on the CI monitoring.

*Response: We revised the methods to provide a more detailed description of cardiac index or CI monitoring.*

*Perioperative procedures and monitoring section*

*“Continuous aortic pressure (MAP), central venous pressure (CVP), cardiac output (CO) and cardiac index (CI) will be monitored with CI is calculated by dividing CO by body surface area.”*

8. The “up to 12 min” is unnecessarily repeated in the first sentence of the cardiac arrest and carbon monoxide experimental protocol section.

*Response: This was corrected*

9. Please provide the rationale for choosing 8 min duration of untreated cardiac arrest.

*Response: Extensive preclinical and clinical research has examined the time-sensitive progression of resuscitation physiology. We first considered the 3 phase model of cardiac arrest which was previously described by Weisfeldt & Becker (JAMA 2002), where immediate defibrillation within a 5-minute arrest period did not benefit from CPR (Neimann et al, Circulation, 1992). For our proposed experiment, we wanted to mimic a real world scenario of sudden cardiac arrest followed by bystander CPR, while also maximizing our chances for return of spontaneous circulation. A number of clinical and preclinical studies have identified that ROSC rates can be optimized in the 5-10 minute no-flow window by including CPR + Defibrillation (Yakaitis et al., Crit Care, 1980; Menegazzi et al, Ann of Emerg Med, 1993; Yu et al, Circulation, 2002). Extending beyond the 10 minute no-flow time appears to decrease chances of ROSC, and the severity of brain injury (outcome of interest) will likely outweigh any benefit from the proposed intervention.*

10. Please provide the reason why epinephrine is chosen as a medication to titrate CI. Epinephrine is well known to affect cerebral perfusion adversely.

*Response: The post-cardiac arrest period is well known to result in reperfusion vasoplegia that would make it very difficult to initiate inodilator therapies (dobutamine, milrinone, etc.) without causing hemodynamic collapse. It is true that during ACLS, preclinical studies have identified that bolus dose (30 mcg/kg) epinephrine can reduce cerebral perfusion (Ristago, Crit Care Med, 2007), but the data is mixed, but our lab has found that lower dose (20 mcg/kg) may actually increase cerebral perfusion (Mavroudis et al, Crit Care, 2020). Post-arrest titration of an epinephrine effusion will allow us to augment cardiac output, while also avoiding excessive vasoplegia. It is unclear if epinephrine infusions, at lower continuous doses, negatively impact cerebral perfusion.*

11. There are two sentences describing the timing of randomization: “All subject animals will be randomized to one of three groups prior to arrival.” and “After successful resuscitation, the animals will be randomly divided into three groups:”

*Response: We revised to the following below*

*Material and methods under cardiac arrest and carbon monoxide experimental protocol*

*“After successful resuscitation, the animals will be randomly divided into four groups: (1) CPR: Animals in this group will only receive CPR without CO upon ROSC; (2) CPR and CO: Cardiac arrest followed by CO treatment; (3) Sham: The same operation but no cardiac arrest or CO being given; (4) CO alone: Will be the same as Control except CO will be administrated to assess possible adverse effects of CO alone [****Figure 1****].”*

12. It would be helpful if you clearly described the timing of commencement of CO administration.

*Response: We revised both the methods and Figure 1 to better describe the timing of each intervention so it is clear when CPR is induced followed by when CPR and CO administration occurs. For CO administration, an hour after achieving ROSC, CO (100 ppm) will be administered for 1 hour followed by 1 hour of re-oxygenation for a total post ROSC time of 3 hours.*

*Figure 1: Experimental Protocol and flow diagram of study*

*The following flow diagram will serve as a general framework for this proposed study with corresponding times: The perioperative period will include the induction of anesthesia to allow for the described procedures such as placement of central lines, cMD catheter, etc that typically takes about 2 hr. The induction of cardiac arrest will take about 4 seconds with cardiac arrest being allowed to continue for 8 min before the initiation of CPR with the first defibrillation at 10 min. If no ROSC is achieved, resuscitation will continue for an additional 10 min for a total of 20 min of CPR. Once ROSC is achieved, CO treatment with 100 ppm will be administrated for 1 hr followed by 1 hr of re-oxygenation for a total of 3 hr post-ROSC.*

13. It would be nice if the authors could share their previous experiences of CO administration, such as hemodynamic changes after CO administration.

Response: We revised the paper to cite our work both in the area of CO poisoning and cardiac arrest that demonstrates our expertise in this area.

*“While patients typically manifest symptoms with higher concentrations of CO, our lab has demonstrated cellular dysfunction in our swine model of acute CO poisoning using a relative lower dose (400 ppm) without overt clinical symptoms”*

14. How many hours after ROSC does the experimental protocol end and euthanasia occur?
Please define the primary outcome of this study.

*Response: We revised the methods and Figure 1 to better describe the timing of each key event in our procedure.*

*Material and Methods*

*Figure 1: Experimental Protocol and flow diagram of study*

*“The following flow diagram will serve as a general framework for this proposed study with corresponding times: The perioperative period will include the induction of anesthesia to allow for the described procedures such as placement of central lines, cMD catheter, etc that typically takes about 2 hr. The induction of cardiac arrest will take about 4 seconds with cardiac arrest being allowed to continue for 8 min before the initiation of CPR with the first defibrillation at 10 min. If no ROSC is achieved, resuscitation will continue for an additional 10 min for a total of 20 min of CPR. Once ROSC is achieved, CO treatment with 100 ppm will be administrated for 1 hr followed by 1 hr of re-oxygenation for a total of 3 hr post-ROSC.”*

Figure legend
15. Please spell out all abbreviations within the Sources of Funding section.

*Response: We added the full funding information as recommended*

1. National Institute of Environmental Health Sciences R21ES031243 (Jang)

2. National Heart, Lung, and Blood Institute R01HL141386 (Kilbaugh)

3. National Heart, Lung, and Blood Institute R56HL158696 (Jang)

4. National Heart, Lung, and Blood Institute R01HL166592 (Jang, Baker)

5. Children’s Hospital of Philadelphia Frontier Program (Baker, Ko, Forti, Kilbaugh)

6. National Institute of Neurologial Disorders and Stroke R01NS113945 (Baker, Lewis, Forti, Kilbaugh)

7. U.S. Department of Defense DoD W81XWH-22-1-0887/8 (Baker, Ko, Forti, Kilbaugh)

8. Toyota Way Forward Fund (Baker, Ko, Forti, Kilbaugh)

16. Please revise the figure to include the timing of each intervention.

*Response: We revised the figure caption to include the timing of each intervention.*

**Reviewer #2:** The proposed study is highly relevant with a very well described method. It will allow a very comprehensive evaluation of the effect of CO after cardiac arrest.

1. The submission of the design of experimental study should be encouraged. However, I do not know whether method paper could comply to Plos One Editorial policy, since the journal guidelines state that research articles "must present the results of original research". In order to include original findings, the article should at least present preliminary original data with a natural history of the model in control conditions.

*Response: As editor mentioned, this submission is appropriate for the protocol section.*

7. PLOS authors have the option to publish the peer review history of their article ([what does this mean?](https://journals.plos.org/plosone/s/editorial-and-peer-review-process#loc-peer-review-history)). If published, this will include your full peer review and any attached files.

If you choose “no”, your identity will remain anonymous but your review may still be made public.

**Do you want your identity to be public for this peer review?** For information about this choice, including consent withdrawal, please see our [Privacy Policy](https://www.plos.org/privacy-policy).

Reviewer #1: No

Reviewer #2: No
