# Supplementary material for: Carbon monoxide as a cellular protective agent in a swine model of cardiac arrest protocol
Source: PLoS One. 2024 May 15;19(5):e0302653. doi: 10.1371/journal.pone.0302653 (PMC11095756; doi:10.1371/journal.pone.0302653)
Supplement: Attachment — Submitted filename: PLOS Response Sheet.docx [file pone.0302653.s002.docx]

Thank-you for the comments. In our previous revisions we addressed the issue with overlap for the related to two of our publications listed below which satisfied the reviewer.

1) Reference 47-"Alteration in Cerebral Metabolism in a Rodent Model of Acute Sub-lethal Cyanide Poisoning" -https://doi.org/10.1007/s13181-022-00928-w

2) Reference 29- "Preliminary Research: Application of Non-Invasive Measure of Cytochrome c Oxidase Redox States and Mitochondrial Function in a Porcine Model of Carbon Monoxide Poisoning" - https://doi.org/10.1007/s13181-022-00892-5

We were asked to address additional editor comments listed below.

“The substantial revision to reduce the overlap with previous publications from the same group in Methods is appreciated. However, there is still minor concern.”

Some information is needed to help the readers better understand certain specific methods:

**1) Please provide one or two sentences to describe how CIV redox rates are determined**

We added the following to the optics section:

*Given that each tissue chromophore has a unique wavelength-dependent absorption signature, it is feasible to isolate the CIV influence on the brain's absorption spectrum from other tissue chromophores. We specifically used the UCLn algorithm to compute changes in cerebral oxidized CIV concentration from the measured wavelength-dependent changes in optical attenuation signals between 780–1000 nm wavelengths.*

**2) How much tissue protein is used for Western Blot and for ATP fluorometry**

For western blot:

*Based on previous work we will use a protein concentration of 10 μl per well. All experiments will be performed in duplicates and the local background corrected density values.*

For ATP fluorometry:

*ATP concentrations will be obtained in snap frozen brain tissue samples (ratio of 10 mg of tissue to 100 µl of the assay buffer) using an ATP fluorometric assay kit (Sigma MAK190) with an excitation of 535 nm and emission of 587 nm obtained in a similar manner based on our previous publications [40, 47].*

**3) The wavelengths for ATP fluorometry should be stated.**

We revised the ATP fluorometry to include the wavelengths based on our previous publications. The below is the revised paragraph.

*ATP concentrations will be obtained in snap frozen brain tissue samples (ratio of 10 mg of tissue to 100 µl of the assay buffer) using an ATP fluorometric assay kit (Sigma MAK190) with an excitation of 535 nm and emission of 587 nm obtained in a similar manner based on our previous publications [40, 47].*
